# Highlighting the value of polymyography in childhood onset movement disorders

**DOI:** 10.3389/fneur.2026.1771878

**Published:** 2026-05-28

**Authors:** Raffaella Moretti, Claudia Ravelli, Yara Ahmar, Nathalie Dorison, Marie De Salins, Diana Rodriguez, Kumaran Deiva, Anne Isabelle Vermersch, Minh Hanh Triboulet, Stéphanie Valence, Madeleine Harion, Lydie Burglen, Emmanuelle Apartis, Diane Doummar

**Affiliations:** 1 Department of Clinical Neurophysiology, Armand Trousseau Hospital, AP-HP-Sorbonne Université, Paris, France; 2 Department of Paediatric Neurology, Armand Trousseau Hospital, Referral Centre for Neurogenetic Rare Disease, APHP-Sorbonne Université, Paris, France; 3 Department of Clinical Neurophysiology, Saint-Antoine Hospital and Pitié-Salpêtrière Hospital, APHP-Sorbonne Université, Paris, France; 4 Unité Dyspa, Neurochirurgie Pédiatrique, Hôpital Fondation Rothschild, Paris, France; 5 Sorbonne University, Paris, France; 6 Department of Paediatric Neurology, Armand Trousseau Hospital, AP-HP Sorbonne University Hospital, Paris, France; 7 Service de Neurologie Pédiatrique, Hôpital Armand Trousseau, AP-HP Sorbonne Université, Paris, France; 8 Centre de Référence Maladies Rares "Déficience Intellectuelle de Cause Rare et Polyhandicap", Paris, France; 9 Centre de Référence des Malformations et Maladies Congénitales du Cervelet et Laboratoire de Neurogénétique Pédiatrique, Département de génétique, Hôpital Armand Trousseau, Paris, France; 10 Paris Brain Institute, ICM, Inserm, CNRS, Sorbonne Université, Paris, France

**Keywords:** childhood, dystonia, myoclonus, neurophysiology, paediatric movement disorders, polymyography, tremor

## Abstract

**Introduction:**

Characterizing hyperkinetic Movement Disorders (MD) in children is challenging, particularly when distinguishing tremor from myoclonus. Polymyography (EMG combined with accelerometry) and EEG jerk-locked back-averaging are well-established diagnostic tools in adults but are rarely applied in paediatric population. This study aimed to assess the feasibility and contribution of individualized neurophysiological investigations to the classification of hyperkinetic MD in children.

**Materials and methods:**

We retrospectively reviewed clinical and neurophysiological data from consecutive patients who underwent polymyography over a two-year period being referred for unclear clinical MD phenomenology. A pediatric MD specialist and a neurophysiologist jointly performed evaluations.

**Results:**

56/60 patients were included (four were excluded due to absent MD during recording or lack of cooperation). Myoclonus was the most frequent polymyography diagnosis (55%), followed by tremor (36%). Initial clinical diagnoses were confirmed in 62% of cases: all suspected cases of myoclonus were validated, whereas 48% patients initially diagnosed with tremor were reclassified as having myoclonus. Polymyography revealed additional MD in 18% of patients, most often myoclonus, and supported a neurofunctional aetiology in one case. After the polymyography, symptomatic pharmacological treatment—mainly for tremor and cortical myoclonus—was proposed in 34%. Additional genetic investigations were suggested in 30% of patients.

**Discussion and conclusion:**

Polymyography proved feasible even in young children, including those with intellectual disability. Combined with expertise of an MD specialist, polymyography significantly improves diagnostic accuracy, particularly tremor vs. myoclonus, and guides both aetiologic and therapeutic management. These findings highlight the value of integrating polymyography into paediatric MD evaluation.

## 1 Introduction

Paediatric hyperkinetic movement disorders (MD) include, in order of frequency, tics, dystonia, chorea, myoclonus, tremor, and stereotypic movements. Neurological examination can provide valuable information on anatomical distribution, temporal frequency, regularity, amplitude, and activation conditions of these involuntary movements, but assessing the nature of hyperkinetic MD can be challenging, especially to distinguish myoclonus from tremor.

**Figure 1:**
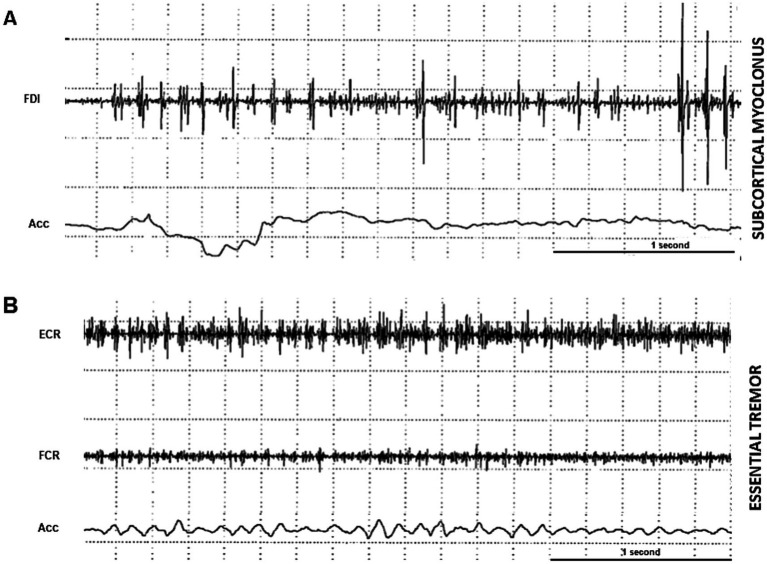
Surface polymyographic recordings of two patients referred for upper limb tremor. In patient **(A)**, polymyography (PMG) demonstrates a fine irregular myoclonus with relatively long bursts during (55–110 ms, always >50 ms) on FDI muscle and irregular arrhythmic signal on Acc placed in the middle finger of the right hand, both corresponding to a subcortical myoclonus mimicking tremor. In patient **(B)**, PMG is consistent with a true tremor, and compatible with an essential tremor (ET); note here the regular rhythmic oscillatory Acc signal (9 Hz) placed on the hand and rhythmic bursts (80 ms) recorded on ECR muscle. ECR, extensor carpi radialis; FCR, flexor carpi radialis; FDI, first dorsal interosseous muscles; Acc accelerometer.

Both myoclonus and tremor have well established definitions: the first is defined as an involuntary, sudden, brief, shock-like ‘jerky’ movement due to muscular contraction (positive myoclonus) or a sudden disruption of ongoing muscle contraction (negative myoclonus) (1); the second is characterized as a rhythmic, involuntary movement of a body part over a joint (2). However, in clinical practice it could be challenging to distinguish these hyperkinetic MD. For instance, it can be difficult to differentiate fine, rapid and rhythmic myoclonic jerks from tremor (“tremor-like” myoclonus, Figure 1). On the contrary, a true tremor may appear irregular and jerky due to voluntary attempts to control the movement, thereby mimicking myoclonus (3). Choreic movements with a jerky component may also resemble myoclonus; dystonia can be difficult to differentiate from compensatory movements when myoclonus, chorea, or tremor impair fine motor skills. Furthermore, these abnormal movements may coexist and it is then essential to determine which MD is predominant. Lastly MD may be associated with other neurological manifestations, further complicating clinical assessment (4).

Therefore, clinical evaluation alone may be insufficient for precise classification, highlighting the need for complementary diagnostic tools.

Clinical neurophysiology, using surface polymyography (EMG) combined with accelerometry and EEG jerk-locked back-averaging (JLBA) method, provides valuable tools for characterizing a broad range of neurological manifestations within the spectrum of hyperkinetic MD. These techniques are particularly useful to differentiate myoclonus from tremor and to provide valuable insights into the pathophysiology and anatomical origin of various hyperkinetic MD (3). For instance, it can delineate the anatomical substrate of myoclonus by identifying a cortical, subcortical, spinal, or peripheral origin (5). Likewise, tremor can be classified into distinct subtypes, including enhanced physiological tremor (EPT), essential tremor (ET), functional tremor (FT), cerebellar outflow tremor (CT), Holmes tremor (HT), dystonic tremor (DT), and parkinsonism-associated tremor (PT) (6). In uncertain or challenging cases suspected to represent a Functional Neurological Symptom Disorder (FNSD), electrophysiological studies may offer unequivocal support for diagnosis.

While clinical neurophysiology is a well-established diagnostic approach in adults (3, 6), its application in paediatric population remains relatively limited and scarcely studied (7). Adult neurophysiology centers can record adolescents, but young children are often excluded. Furthermore, the feasibility of neurophysiological studies in infant and children may be challenging, as young age, behavioral difficulties and/or intellectual disability can impair their ability to understand the examination instructions and perform the required tasks.

If the phenomenology of abnormal movements shows similarities between adolescents and adults, some differences are observed in younger children. Indeed, motor function evolves from birth to young adulthood in line with central nervous system maturation, which may result in atypical clinical presentations, modify MD phenomenology over time, and therefore interfere with the comprehension and description of MD in children.

Additionally, paediatric aetiologies of MD are different from adults, notably genetic disorders are more frequent. Nowadays, rapid advances in genetics have substantially increased the yield of etiological diagnoses in childhood-onset MD. Nevertheless, detailed analyses of phenomenology and pathophysiology remain essential for guiding clinical diagnosis and therapeutic strategies, and therefore continue to benefit from neurophysiological expertise.

The aim of our study is to highlight the contribution of individualized electrophysiological investigation combined to MD expert analysis to the characterization of hyperkinetic MD in children, especially to distinguish myoclonus from tremor.

The primary objective is to determine the feasibility of polymyography in children. The secondary objective is to assess the contribution and accuracy of polymyography combined with clinical MD expert examination to classify the predominant MD and to define generator subtypes.

We present the results of a monocentric study conducted at a Reference Centre for Paediatric Movement Disorders.

## 2 Materials and methods

We retrospectively reviewed clinical and neurophysiological data from consecutive patients who underwent polymyography (EMG combined with accelerometry) between October 2023 and October 2025 at our paediatric neurophysiology department.

Children were referred by neuropaediatricians—whether specialized in MD or not—for (i) confirmation of the type of hyperkinetic MD, (ii) characterisation of unclear phenomenology, (iii) determination of the neurophysiological origin of myoclonus and tremor, especially in patient with identified genetic aetiology, (iv) distinguishing organic versus FNSD.

We also reviewed personal and familial medical histories, together with preclinical findings, particularly metabolic and hormonal analyses as well as MRI.

### 2.1 Polymyography studies in our centre: examination techniques

Neurophysiological examination was performed in the presence of a paediatric MD specialist and a neurophysiologist with expertise in MD in both paediatric and adult populations. It included a detailed clinical analysis of MD.

Polymyography and accelerometry were performed simultaneously, with recordings obtained from surface EMG of the relevant muscles and an accelerometer (Acc). The accelerometer, a piezoelectric sensor, was attached to the body part involved in the abnormal movement (polygraphy methods are detailed in Supplementary data).

Muscle activity was assessed at rest, during voluntary action, intention, or specific tasks (neurological motor tests, writing, drawing, water transfer, and fine motor skills such as pen blocking or toys manipulation) adapted to the child’s age and psychomotor development. The main features analysed included rhythmicity of EMG bursts and accelerometric signals, EMG burst duration, spatiotemporal organization of muscle contractions, and responsiveness to stimuli (light touch, pinprick, muscle stretch) or activation paradigms (mental activation, voluntary action, distraction). Competitive voluntary motor tasks were also used to assess variability and distractibility of the MD. This methodology allowed precise characterization of the type of MD and clarification of its anatomical substrate.

These techniques are particularly useful to differentiate myoclonus from tremor. Duration of the myoclonus EMG discharge is typically ≤100 ms and never exceeds 260 ms. Tremor is an oscillatory movement of a body part with a frequency generally ranging from 3 to 12 Hz. Rhythmic myoclonus can mimic tremor but accelerometry and EMG patterns are regular in tremor, showing a distinct main frequency, whereas they are irregular in myoclonus and have a wider frequency distribution; moreover, single bursts can be very brief (especially if myoclonus originates from neocortex).

**Figure 2:**
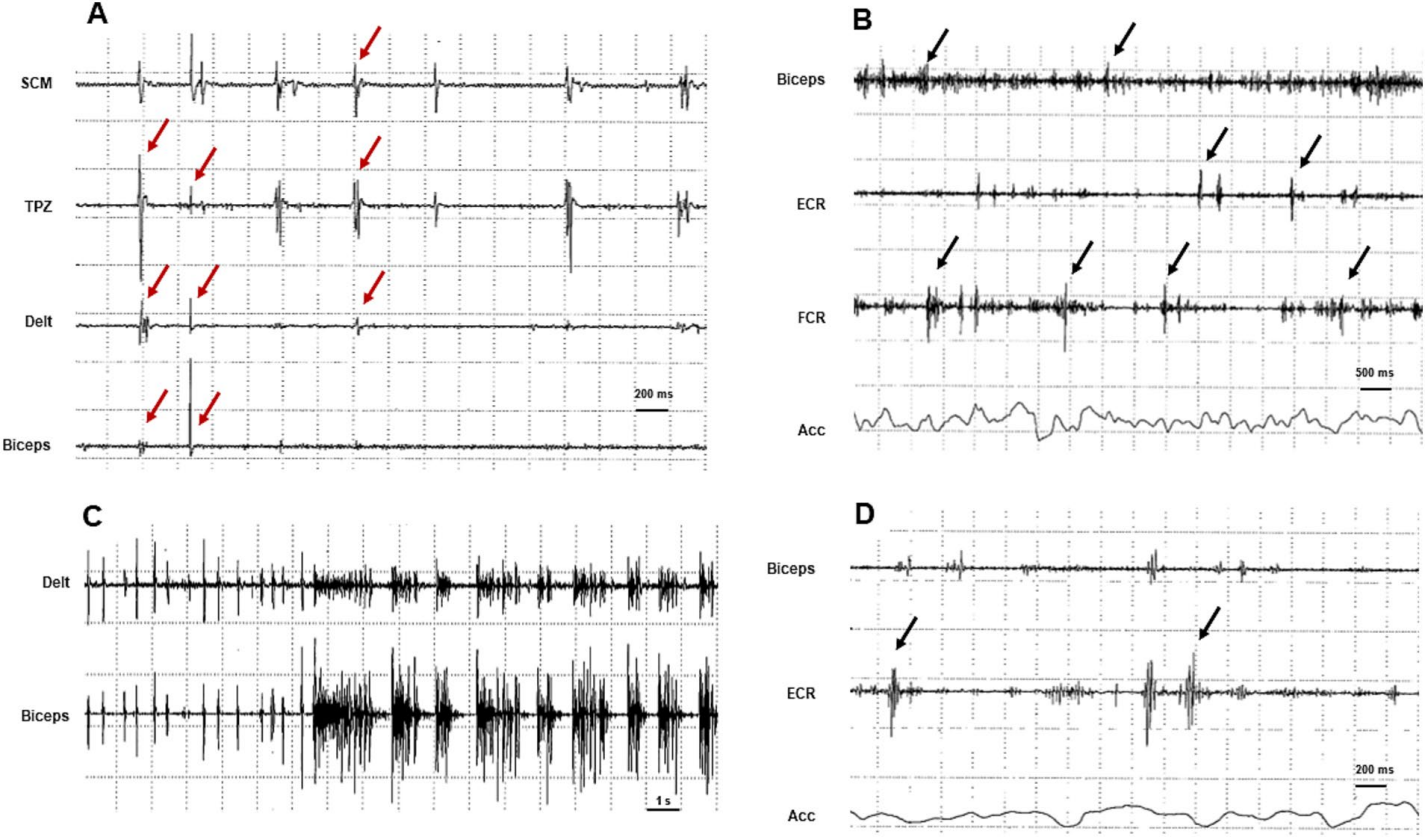
Polymyographic and Acc recordings of cortical myoclonus **(A,C)** compared to subcortical myoclonus **(B,D)**. In **(A)**, polymyography demonstrates repetitive, very brief (13 to 24 ms) myoclonic bursts, synchronous in several muscles (red arrows) recorded over the neck and upper limb muscles; in **(C)**, note the temporal evolution of these cortical myoclonic jerks into a clonic seizure. In **(B,D)**, polymyography shows irregular, erratic jerks (black arrows) in the forearm and hand muscles, of longer duration (82 ms, 92 ms, and mainly over 50 ms) than in **(A)**. SCM, sterno-cleïdo-mastoideous; TPZ, trapezius; ECR, extensor carpi radialis; FCR, flexor carpi radialis; Acc, accelerometer.

If myoclonus was identified, patients underwent conventional coupled EEG/EMG recordings, and a JLBA method was applied when direct visual inspection of the raw EEG–EMG signals failed to reveal clear cortical events correlated with myoclonus. Cortical myoclonus is characterized by brief jerks (<50 ms) of focal or multifocal distribution and either occur spontaneously or are triggered by various distal somesthetic stimuli such as touch, pinprick or muscle stretch. As it propagates along the corticospinal pathway, cortical myoclonus activates rostral muscles first and then the more caudal segments. Subcortical myoclonus has a more heterogeneous polymyographic recording, EMG burst durations ranges from 25 to 250 ms, with erratic timing in the involved muscles, and stimulus sensitivity is unusual (Figure 2) (3, 8).

In addition, polymyography supported the diagnosis of FNSD by providing objective evidence of inconsistencies in motor patterns. Features that characterize FNSD movements are: (i) acute onset and sudden resolution; (ii) spontaneous periods of remission; (iii) inconsistent movement patterns (amplitude, frequency and distribution) and (iv) marked reduction of MD with distraction, the latter two being well demonstrated with polymyography (9).

At the end of the neurophysiological/clinical exam, the hyperkinetic MD were classified according to the predominant MD and generators subtypes were defined, when possible.

Polymyographic features were compared with clinical classification in order to evaluate the role of polymyography in the identification of MD and its contribution to diagnosis.

*Ethical*: This study was performed in accordance with French regulations and the principles of the Declaration of Helsinki. This work obtained the approval of the ethical committee of the French Paediatric Society.

## 3 Results

We retrospectively included 60 patients with suspected tremor, myoclonus, or unclassified hyperkinetic MD. Of these, two were excluded because polymyographic assessment could not be performed, and two due to the absence of abnormal movements during neurophysiological recording.

A total of 56 patients were analysed, including 34 males and 22 females. The mean age at onset of hyperkinetic MD was 6 years (range: 1 month to 15 years), with a mean age at polymyographic evaluation of 11.6 years (range: 18 months to 18 years). The median duration of abnormal movements was 5.6 years (range: 1 month to 14 years).

Children were referred to confirm the initial clinical diagnosis of predominant myoclonus or tremor (41/56) or to characterize the type of MD in the absence of a clinical diagnosis or in presence of combined MD (meaning the association of MD) with the impossibility to identify the prevalent one (15/56). Among the patients with an established rare neurogenetic disorder, clinicians also asked to refine the MD phenotype in relation to the underlying genetic variant (17/56). Two patients were referred to confirm suspected FNSD.

Regarding the classification of abnormal movement, patients were categorized according to the predominant MD. For most of the children, clinical examination by MD expert combined with polymyography allowed to identify a predominant MD (primarily tremor or myoclonus); in some patients, an additional MD was identified and it was clearly less prominent (for example, very sparse myoclonus in addition to tremor, mild dystonia in addition to myoclonus, etc.).

**Figure 3:**
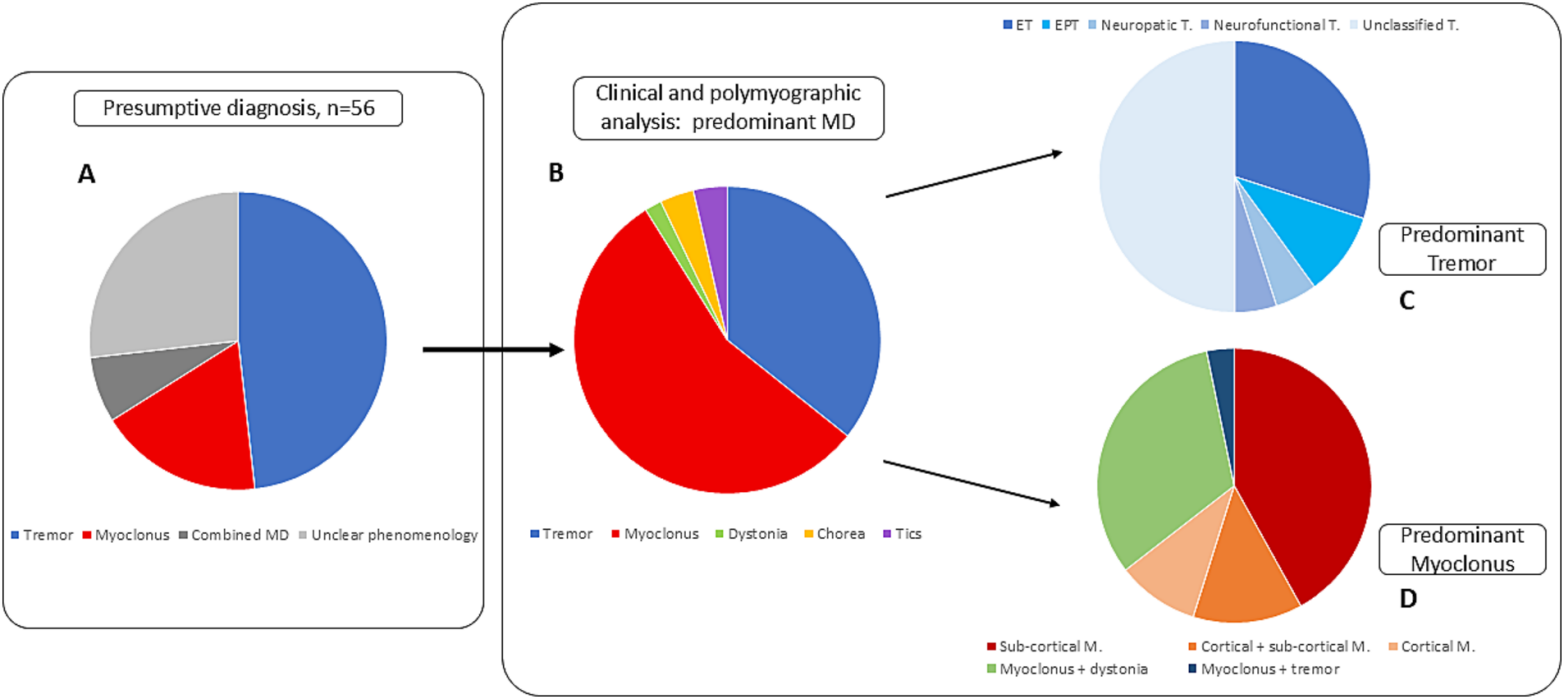
Changes between presumptive diagnosis **(A)** and final diagnosis **(B)** (*n* = 56). Detail of polymyography results in the tremor **(C)** and myoclonus subgroup **(D)**. ET, Essential Tremor; EPT, Exagerated Physiologic Tremor; T, Tremor; MD, movement disorder; M, myoclonus.

Among the 41/56 patients with a presumptive clinical diagnosis, 27 were classified as tremor, 10 as myoclonus, and 4 as combined MD (Figure 3A). Concordance between the initial clinical diagnosis and the polymyographic diagnosis was observed in 26 of 41 cases (62%). The clinical diagnosis of myoclonus was confirmed in all instances whereas tremor diagnoses showed lower concordance: 13 of 27 patients (48%) were reclassified as having myoclonus.

Among the 15/56 patients without an established clinical diagnosis of MD, polymyography revealed isolated myoclonus in six cases (including two tremor-like, two cortical and subcortical, and one subcortical myoclonus), myoclonus combined with other MD in six cases (four with dystonia, one with tremor, and one with chorea), tics in two cases and tremor in one case.

**Figure 4:**
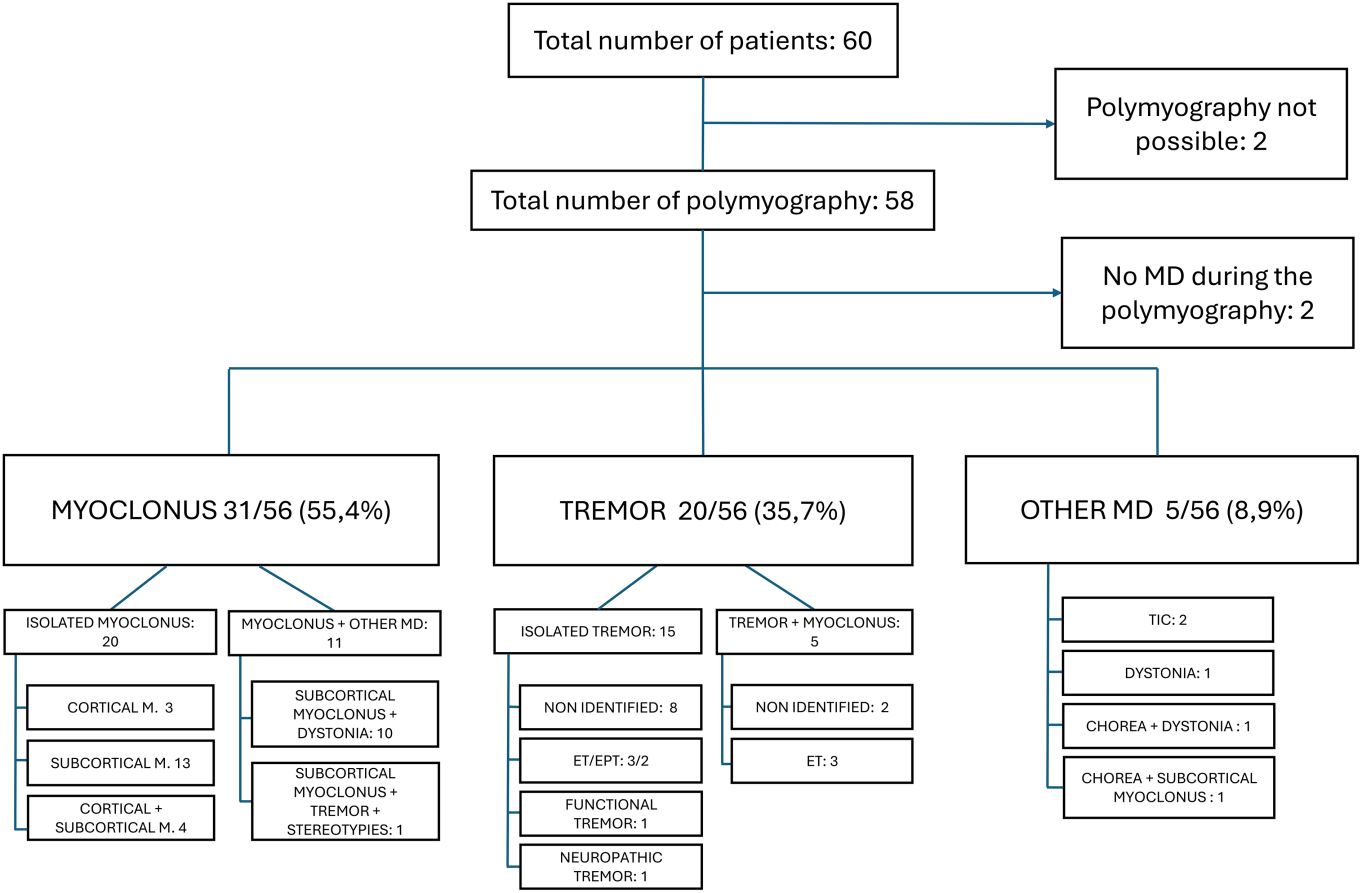
Final MD classification and generators subtypes of predominant MD after combined clinical and polymyography analysis. M. myoclonus; ET, essential tremor; EPT, exaggerated physiologic tremor.

The final diagnosis after polymyography combined with clinical evaluation of expert in MD in all 56 patients is resumed in Figures 3B–D, Figure 4.

*Myoclonus* was diagnosed in 31 patients (55.4%), isolated in 20 cases—predominantly of subcortical origin (in 13 patients), cortical in three patients, and both cortical and subcortical in four. Predominant myoclonus was combined to other MD in 11 patients, most frequently dystonia (10/11) (Figures 3D, 4).

*Tremor* was diagnosed in 20 of 56 patients (35.7%) and was isolated in 15 cases. Essential tremor (ET) and enhanced physiological tremor (EPT) were identified in, respectively, three and two patients while neuropathic tremor was diagnosed in one patient and functional tremor in another. The remaining eight cases of isolated tremor were unclassified. Predominant tremor was associated with myoclonus in five patients: in three cases, the diagnosis of ET was established; in the two others, a genetic diagnosis was already established (pathogenic variants in the *MTHFD1* gene and *DNJAC6 gene*, without parkinsonism at age 6 years) (Figures 3C, 4).

*Other MD* were identified in 5 out of 56 patients (9%): tics in two, isolated dystonia in one, chorea in two, combined with dystonia in one patient and with myoclonus in another (Figures 3B, 4).

Combined clinical and polymyographic assessment identified an additional MD not clinically recognized by the referral doctor in 10 out of 56 patients (18%): myoclonus in five, mild dystonia in three and tremor in two cases.

A genetic aetiology was previously established in 17 of 56 patients (30%). Following the neurophysiological examination, next-generation sequencing (NGS) analysis was proposed to the referring physician in 17 patients, resulting in six negative findings, two variants of uncertain significance (VUS), and pending results in the nine remaining patients.

Following clinical and polymyographic evaluation, symptomatic pharmacological treatment—mainly for tremor and cortical myoclonus—was proposed to the referring physician in 21 patients (34%). A partial clinical response was observed in 6 patients, while in the others, treatment efficacy could not yet be determined due to the short follow-up period.

### 3.1 Illustrative case 1

A 14-year-old girl was referred to our neurology department for evaluation of a tremor.

She was born after an uneventful pregnancy and delivery, and exhibited normal psychomotor development.

A “tremor” was first noted at 8–9 years of age, associated with mild handwriting difficulties. The MD caused no major disability or pain and was exacerbated by emotion and stress. Her schooling has been normal.

The medical history was notable for familial thyroid disease: her mother and maternal grandmother were followed for Hashimoto’s thyroiditis treated, respectively, with levothyroxine and thyroidectomy and both presented a postural “tremor.” The patient’s weight has remained around −2 SD and her height around −1 SD.

Neurological examination by MD expert revealed a predominantly postural upper limb myoclonus, both proximal and distal. Rapid frequency and small amplitude of myoclonus jerks gave a « tremor like » appearance. Myoclonus was not modified by action. At rest, occasional jerks were observed. No other neurological abnormalities were detected. Polymyography performed at age 13 showed rare, erratic distal myoclonus at rest, markedly aggravated by posture and minimally by action, with a frequency between 7 and 10 Hz and a duration of 55–110 ms. These myoclonic discharges were occasionally rhythmic, thereby mimicking a tremor. In addition, polymyographic features of myoclonus was typical of subcortical myoclonus origin as shown in Figure 1A.

Polymyography led to reclassification of symptoms as isolated subcortical myoclonus without dystonia, rather than tremor.

Standard laboratory tests, including thyroid function, were normal.

Genetic testing and brain MRI were recommended and polymyography was proposed for the patient’s mother to further investigate her reported “tremor.”

The patient’s myoclonus was not disabling, and no treatment was proposed.

### 3.2 Illustrative case 2

A 17-year-old girl was referred to our paediatric neurology department for evaluation of intellectual disability and epilepsy.

She was born in Tibet to non-consanguineous parents after an uneventful pregnancy and delivery. Early developmental milestones were delayed: she achieved head control at 9 months and independent sitting at 18 months old.

At 2½ years old, following an episode of encephalitis of unspecified aetiology in India, she developed epilepsy. She achieved autonomous walking at 3½ years old and exhibited an obvious language delay.

She arrived in France at the age of 14 years. On examination, she presented with moderate intellectual disability (jovial behavior, comprehension difficulties, and limited verbal expression), but her neurological examination was otherwise unremarkable. Focal seizures persisted and her antiseizure treatment was adjusted. Brain MRI revealed bilateral frontal atrophy.

At 16 years of age, she experienced a second acute neurological episode following a viral infection, characterized by cerebellar ataxia, “tremor” and hyperkinetic movement difficult to categorize. There was no subsequent recovery. Brain MRI demonstrated the occurrence of bilateral T2 and FLAIR hyperintensities in the thalami and corticospinal tracts.

At 17 years of age, she maintained a jovial behavior, communicated by stereotyped small sentences, and exhibited unsteady walk with persistent cerebellar ataxia. Distal and proximal myoclonus was observed at rest, exacerbated by action. She also experienced paroxysmal worsening of myoclonic jerks that prevent her from being able to walk. Her focal epileptic seizures were controlled by lacosamide and topiramate. EEG showed rare sporadic frontal discharges with no correlation to myoclonus on EMG.

Polymyography revealed predominant cortical myoclonus affecting the upper limbs, associated with rare subcortical myoclonus (Supplementary Figure 1, on Supplementary data). These findings allowed to determine the neurophysiological origin of myoclonus, which could not be identified clinically, and supported treatment modification: lacosamide was replaced by lamotrigine.

Whole-genome sequencing identified a pathogenic heterozygous *de novo* variant in *DHDDS* gene, coding for an enzyme required for biosynthesis of several classes of glycoproteins, consistent with her clinical phenotype.

## 4 Discussion

Our retrospective study aimed to evaluate the feasibility and the input of polymyography combined with clinical MD expert examination on the characterization of hyperkinetic movement disorders and on guiding both etiological and therapeutic approaches in 56 children.

In the existing literature, studies employing polymyography in paediatric populations remain scarce. To date, only Canavese et al.’s study (7) reported the results of a combined clinical and polymyographic retrospective analysis of 61 children with MD, predominantly dystonia, as well as myoclonus and tremor.

Additional cases were described in the study by Everlo et al. (6), which examined the role of neurophysiology in distinguishing myoclonus from tremor in a cohort of 773 patients with a mean age of 49 years (range: 0–91 years, SD: 23), without specific details on paediatric cases.

Callister et al. (10) reported a neurophysiological study of 262 patients referred for evaluation of suspected myoclonus. However, this cohort consisted only of adult patients.

To our knowledge, our study represents the first paediatric cohort specifically addressing the complexity of differentiating myoclonus from tremor, which is frequently employed as common clinical descriptor of distal hyperkinetic MD.

First of all, our study demonstrates that clinical neurophysiology using EMG and accelerometry is a non-invasive and feasible evaluation, even with very young children—the youngest patient studied being 18 months old—as well as with children presenting mild to moderate intellectual disability and/or having behavioral problems. Examinations were adapted and tailored to ensure that the child felt comfortable and confident, notably through the integration of play-based activities. It is interesting to note that in several cases polymyography can provide accurate diagnostic information even when child cooperation is only partial.

Polymyography allowed characterization of MD in all patients recorded, except for the two in whom no MD was observed during the neurophysiological examination due to the paroxysmal nature of their symptoms. This finding highlights the importance of selecting patients with continuous or frequent abnormal movements (occurring several times per day) to optimize diagnostic yield.

Our retrospective study demonstrates that clinical classification of MD remains challenging, as evidenced by the comparison between purely clinical evaluation and the assessment combining expert MD clinical analysis and polymyographic study. Among the 41 patients with an initial presumptive clinical diagnosis, polymyographic findings were concordant in 26 cases (62%). Comparable concordance rates have been reported by Canavese et al. (7) and Everlo et al. (6) (respectively around 70 and 54%).

Notably, all 10 clinically suspected cases of myoclonus were confirmed by polymyography. In contrast, tremor diagnoses were frequently reclassified, with 13 of 27 patients (48%) ultimately identified as having myoclonus.

Remarkably, the frequency of MD types in our paediatric series differs from adult cohorts: myoclonus was the most frequent MD, while tremor is the most common movement disorder in adults. In the large cohort reported by Everlo et al. (6), which included 773 adults and children referred for MD classification, myoclonus accounted for only 18% of cases compared to 55.4% in our series, whereas tremor represented 72% versus 35.7% in our series. In this study, MD classification was not stratified according to age and the mean age was 49 years old. In the Callister et al. (10) cohort of 262 adult patients referred for consideration of myoclonus, 59% was reclassified after polymyographic study, mainly having tremor or FNSD.

Consistent with previous studies, combined assessment by a MD specialist and polymyography enabled the identification of an additional clinically unrecognized movement disorder in 18% of our patients. Myoclonus represented the movement disorder most frequently missed.

Once MD was characterized by polymyography, identification of underlying anatomical and physiological substrates provided valuable guidance for both diagnostic refinement and therapeutic management (Illustrative case 1 and 2).

*Regarding the 31 children with predominant Myoclonus*, in contrast to adult patients, only cortical and subcortical myoclonus were observed. None had spinal nor brainstem generated myoclonus. Cortical myoclonus (isolated or associated with subcortical myoclonus) was poorly detected by clinical examination and was not identified by routine EEG, which did not record spikes preceding myoclonic jerks on EMG. In most of our patients with suspected cortical myoclonus, JLBA could not be performed due to lack of cooperation, but polymyographic findings were characteristic of cortical origin and permitted to precise the myoclonus origin. Notably, epilepsy was associated in 6 of 7 patients with cortical myoclonus. Following this polymyographic evaluation, new antiseizure medications were proposed, most frequently lamotrigine.

*Regarding the 20 children with predominant tremor*, clinical examination identified postural and action tremor in all patients. Unlike adults’ cohorts (11), additional rest tremor was observed in only two children of our series: one having functional tremor and the other presenting tremor of unspecified origin. ET was the most frequent subtype, identified in six children (30%), frequency consistent with the literature (from 5 to 30% in paediatric series) (12). Notably, in three of the six ET patients, predominant tremor was associated to mild myoclonus, as previously documented by Piarroux et al. (12). The reason for this association remains uncertain, possibly related to developmental aspects of brain maturation in childhood. Symptomatic treatment with propranolol was proposed, proving beneficial in most cases.

In 10 patients with tremor, the exact origin of MD could not be determined. Seven of these patients had a rare genetic disease, including two with a metabolic disorder (vitamin B12 deficiency and vitamin B6-dependent epilepsy) and one with Charcot–Marie–Tooth disease due to a duplication in 17p11.2 containing *PMP22*. In this last patient, the clinical and neurophysiological characteristics of the tremor (5 Hz postural and action proximal tremor) were atypical for a tremor associated to hereditary polyneuropathy, which is mainly known as a mild postural distal upper limb tremor (13–15), but no other aetiology was found, namely no cerebellar nor dystonic pathology.

Interestingly, in our series, the diagnosis of FNSD was suspected only in two patients having tremor and they were both reclassified as having EPT, as often occurred in the Everlo et al. cohort (6).

A diagnosis of neurofunctional tremor was supported by polymyography in only one patient referred for suspicion of dystonic tremor. In Everlo et al. cohort, (6) FNSD represented 25% of cases. Otherwise, studies combining clinical and polymyographic analysis remain very scarce, making comparisons between children and adults difficult. The low frequency of FNSD in our series may be explained by a bias related to the small simple size. However, it is recognized that the frequency of neurofunctional tremor is lower in children than in adults if we compare series of tremor diagnosed by clinical assessment (16, 17).

Remarkably, distractibility without additional criteria of FNSD was also observed in two patients (one with a 4 Hz tremor of undetermined aetiology and the other with a mobile dystonia of the right upper limb slowly worsening), but clinical and polymyography evaluation strongly supported an organic MD diagnosis. Furthermore, in dystonic disorders, the coexistence of dystonia and FNSD has been reported, for instance in Dopa Responsive Dystonia (18).

Finally, in the 17 patients having already a rare genetic diagnosis (30%), expert MD evaluation combined to polymyography provided detailed characterization of MD phenotypes that are scarcely documented, thereby supporting the use of symptomatic treatments when appropriate.

The main limitations of our study include the relatively small sample size, the monocentric basis and the retrospective design. These limitations could have biased the results. Consequently, prospective evaluations in larger paediatric cohorts may provide greater impact and stronger evidence. Another limitation concerns neurophysiology analysis: in our laboratory, rhythmicity (the hallmark feature of tremor) is primarily based on visual inspection of Acc and EMG signals. It would be interesting to complement visual analysis with power spectral analysis (PSD) of muscle discharge, which has recently been reported to help differentiating tremors from myoclonus (19). Finally, due to the short follow up, we could not provide the results of genetic tests and therapeutic outcomes of all cases.

## 5 Conclusion

This retrospective paediatric study demonstrates that polymyography is a feasible and non-invasive tool that contributes significantly to the characterization of hyperkinetic MD. Beyond diagnostic refinement, it also supports therapeutic decision-making, including the proposal of symptomatic treatments, and guidance for genetic testing. The collaborative approach between expert clinicians in MD and neurophysiologists strengthened diagnostic accuracy and overall quality of electrophysiological interpretation. These findings highlight the value of integrating polymyography into paediatric MD evaluation.

## Data Availability

The original contributions presented in the study are included in the article/Supplementary material, further inquiries can be directed to the corresponding authors.
