# Supplementary material for: Highlighting the value of polymyography in childhood onset movement disorders
Source: Front Neurol. 2026 May 28;17:1771878. doi: 10.3389/fneur.2026.1771878 (PMC13253225; doi:10.3389/fneur.2026.1771878)
Supplement: Supplementary file 1 [file Data_Sheet_1.PDF]

Supplementary Material

1 Supplementary Data

Polymyography and accelerometry: simultaneous recording of movement with an accelerometer (Acc) and surface EMG of relevant muscles was performed by using a Nihon Kohden EMG device owing 4 amplifiers. The piezoelectric sensor Acc was attached to the body part affected by the abnormal movement. Surface EMG was obtained with Ambu Neuroline solid gel electrodes placed in pairs over the muscle bellies after mild skin exfoliation. Band pass filtering was set at 20-500 Hz for EMG and at 0.5-100 Hz for Acc. For each patient, 3 to 7 relevant muscles were selected following a careful clinical analysis.

Conventional EEG: conventional EEG was mandatory for myoclonus to identify epileptiform discharge and possibly an epileptic syndrome, including facilitation methods such as photic stimulation or noise, in order to induce reflex myoclonus or startle. The effect of different mental states namely sleep was systematically explored. A simultaneous EMG recording was performed to analyze EEG/EMG temporal correlations. Cortical spikes were considered as linked with jerks if it occurred 10-20 ms before jerks in arms and 20-30 ms before jerks in legs (depending on patient size), the delay being compatible with the fast corticospinal pathway conduction time.

EEG jerk-locked back-averaging method (JLBA): as cortical spikes may be out of sight due to their low amplitude, an averaging of EEG traces triggered by EMG myoclonus (JLBA) was systematically performed in cases of normal EEG or when direct visual inspection of the raw EEG-EMG signal failed to reveal clear correlation between cortical events and myoclonus. The presence of a short latency cortical positivity preceding myoclonus onset with the cortico-muscular latencies described above provided an electrophysiological signature of cortical myoclonus.

Cortical myoclonus (CM) clinically manifests with jerks affecting the distal limbs or the face. It is often action-induced, positive and negative, and sensitive to somatosensory stimuli (8). CM is characterized by short length jerks (<50 ms). Subcortical-non segmental myoclonus represents an heterogeneous entity : abnormal activity may originate from various sites end circuits ranging from the basal ganglia to the spinal cord and then transmitted to ascending and descending motor pathways. Typically EMG discharges are longer than in CM and additional neurophysiologic testing exclude the presence of a cortical generator. The more common pediatric subtype of this subcortical myoclonus is characterized by an erratic temporo-spatial distribution with jerks mostly elicited by muscle activation but rarely stimulus induced. Myoclonus dystonia syndrome belongs to this category (1)

2 Supplementary Figures and Tables

Supplementary figure 1. Polymyographic recordings of patient described on illustrative case 2. The teenager was referred for characterization of myoclonus. PMG demonstrates a fine rapid irregular rhythmic myoclonus with very brief bursts (13 to 24 ms) on ECR, FCR and FDI muscle and irregular arrhythmic signal on Acc placed in the middle finger of the right hand. Myoclonic bursts are often

synchronous between several muscles (red arrows), and/or followed by a brief inhibition (black arrow). These PMG characteristics correspond to a cortical myoclonus.

Abbreviations: PMG, polymyography; ECR, extensor carpi radialis; FCR, flexor carpi radialis; FDI, first dorsal interosseous muscles; Acc, accelerometer.
