# Supplementary figures and images for: Highlighting the value of polymyography in childhood onset movement disorders
Source: Front Neurol. 2026 May 28;17:1771878. doi: 10.3389/fneur.2026.1771878 (PMC13253225; doi:10.3389/fneur.2026.1771878)

## Slide 1
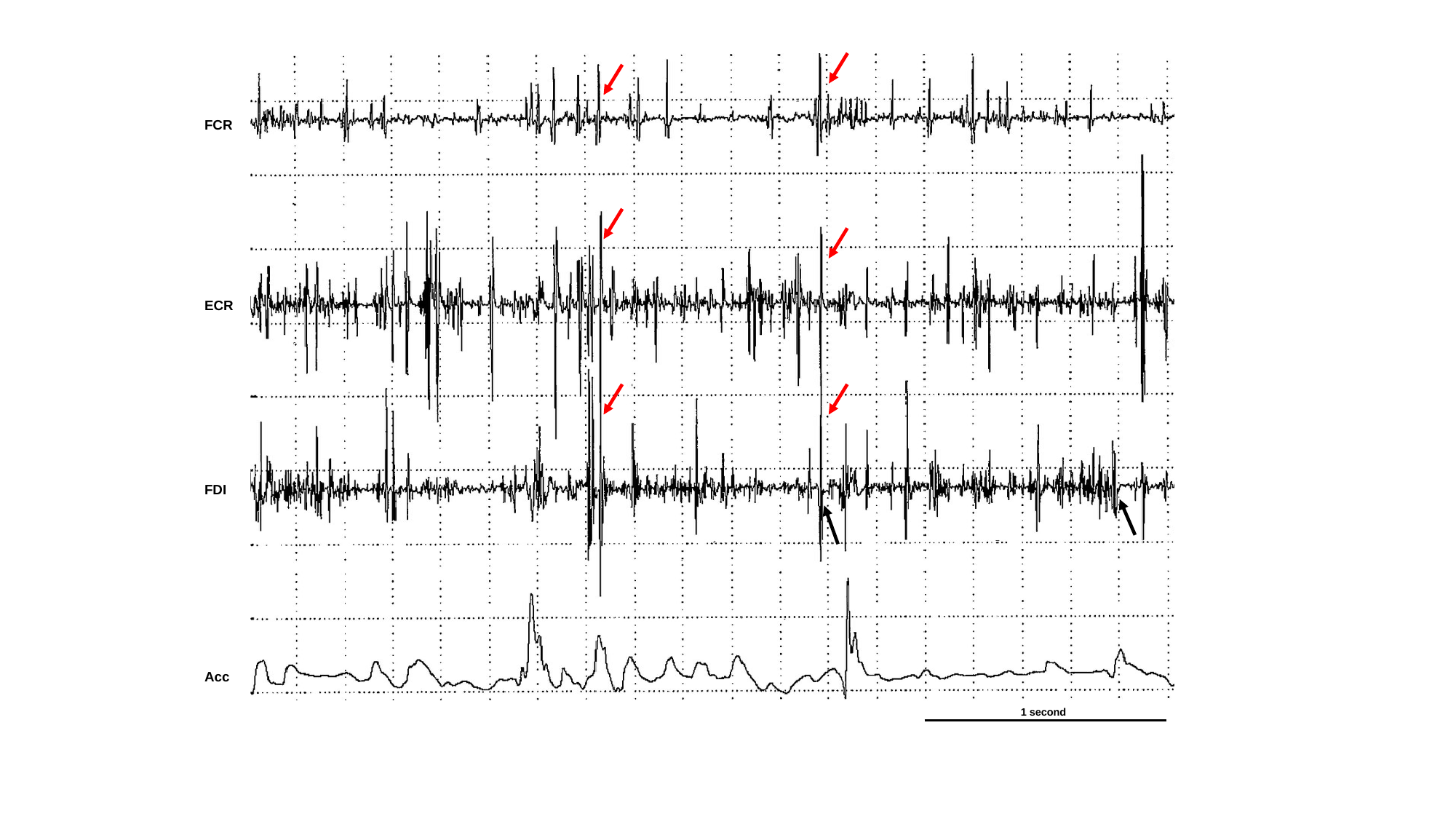

FCR
ECR
FDI
Acc
1 second

Supplement: Supplementary file 2 [file Presentation_1.PPTX]
